# Association between enlarged perivascular spaces in basal ganglia and cerebral perfusion in elderly people

**DOI:** 10.3389/fneur.2024.1428867

**Published:** 2024-07-05

**Authors:** Simeng Wang, Shuna Yang, Dong Liang, Wei Qin, Lei Yang, Xuanting Li, Wenli Hu

**Affiliations:** ^1^Department of Neurology, Beijing Chao-Yang Hospital, Capital Medical University, Beijing, China; ^2^Department of Neurology, Affiliated Hospital of Heze Medical College, Heze, Shandong, China

**Keywords:** enlarged perivascular spaces, cerebral small vascular disease, cerebral perfusion, cerebral blood volume, CT perfusion imaging

## Abstract

**Background and objective:**

Enlarged perivascular spaces in basal ganglia (BG-EPVS) are considered an imaging marker of cerebral small vessel disease (CSVD), but its pathogenesis and pathophysiological process remain unclear. While decreased cerebral perfusion is linked to other CSVD markers, the relationship between BG-EPVS and cerebral perfusion remains ambiguous. This study aimed to explore this association.

**Methods:**

Elderly individuals with severe BG-EPVS (*n* = 77) and age/sex-matched controls (*n* = 89) underwent head CT perfusion imaging. The cerebral perfusion parameters including mean transit time (MTT), time to maximum (TMAX), cerebral blood flow (CBF), and cerebral blood volume (CBV) were quantitatively measured by symmetric regions of interest plotted in the basal ganglia region. Point-biserial correlation and logistics regression analysis were performed to investigate the association between BG-EPVS and cerebral perfusion.

**Results:**

There were no significant differences in MTT, TMAX, or CBF between BG-EPVS group and control group. CBV was significantly lower in the BG-EPVS group (*p* = 0.035). Point-biserial correlation analysis showed a negative correlation between BG-EPVS and CBV (*r* = −0.198, *p* = 0.011). BG-EPVS group and control group as the dependent variable, binary logistics regression analysis showed that CBV was not an independent risk factor for severe BG-EPVS (*p* = 0.448). All enrolled patients were divided into four groups according to the interquartile interval of CBV. The ordered logistic regression analysis showed severe BG-EPVS was an independent risk factor for decreased CBV after adjusting for confounding factors (OR = 2.142, 95%CI: 1.211–3.788, *p* = 0.009).

**Conclusion:**

Severe BG-EPVS is an independent risk factor for decreased CBV in the elderly, however, the formation of BG-EPVS is not solely dependent on changes in CBV in this region. This finding provides information about the pathophysiological consequence caused by severe BG-EPVS.

## 1 Introduction

Perivascular spaces (PVS), also known as Virchow-Robin Spaces, are the tiny spaces in the brain’s parenchyma that surround small arteries and veins and are enclosed by the pia mater. They function as part of the brain’s lymphatic system. PVS will dilate with the accumulation of interstitial fluids. Enlarged perivascular spaces in the basal ganglia (BG-EPVS) is one of the imaging manifestations of cerebral small vascular diseases (CSVD). Studies have shown that severe BG-EPVS is closely associated with a variety of neurological diseases, including cognitive impairments characterized by declines in information processing and executive functions (1), as well as gait disturbances marked by lower gait speed, shorter stride length and reduced stride speed (2, 3). However, the underlying pathogenesis of BG-EPVS and pathophysiological consequences caused by BG-EPVS are still unclear.

Cerebral ischemia is considered to be one of the mechanisms of CSVD (4). Previous studies demonstrated a relationship between other CSVD markers and cerebral blood flow (CBF), such as the severity of white matter hyperintensity (WMH) (5), increased number of cerebral microbleed (CMB) (6), and presence of lacunar infarction (7) are associated with reduced CBF. However, the association between BG-EPVS and cerebral perfusion is still unclear.

Miles (8) first described CT perfusion imaging (CTP), which is an excellent functional neuroimaging technology that can detect brain microcirculation abnormalities, so as to reflect the areas with changed perfusion and metabolic status. CT scanners and CTP post-processing software are extensive and more economical than other functional brain imaging technologies. Head CT perfusion was firstly detected through the intracranial vascular system by monitoring iodized contrast agents. It can quantify and display various perfusion parameters, such as CBF, cerebral blood volume (CBV), mean transit time (MTT), time to maximum (TMAX).

Therefore, we aimed to explore the association between BG-EPVS and cerebral perfusion by head CTP, and to further elucidate the pathogenesis of BG-EPVS and pathophysiological consequences caused by BG-EPVS.

## 2 Patients and methods

### 2.1 Study population

In this study, 77 elderly patients with severe BG-EPVS in Beijing Chao-Yang Hospital, Capital Medical University from August 2020 to March 2023 were selected as the BG-EPVS group, including 51 males and 26 females, aged 60–88(73.95 ± 6.81) years. Another 89 age and sex matched patients with none or mild BG-EPVS were selected as the control group, including 62 males and 27 females, aged 60–90 (72.96 ± 6.74) years.

Inclusion criteria for all subjects: (1) Aged 60 or above; (2) Head CTP and MRI examination had been completed; (3) Patients agreed to participate in the study. Exclusion criteria: (1) Acute cerebrovascular disease diagnosed by head MRI; (2) According to the TOAST classification, the patients were previously diagnosed as non-small arteriolar occlusive cerebral infarction; (3) Magnetic resonance angiography or CT angiography or head and neck vascular ultrasound showed patients with ≥50% of intracranial and/or extracranial large blood vessel stenosis; (4) Patients with neurodegenerative diseases such as Parkinson’s disease, dementia, multisystem atrophy, nervous system infections, demyelinating diseases, brain trauma, intracranial space occupying lesions, poisoning, brain tumors, seizures, radiation encephalopathy, genetic metabolic diseases and other neurological diseases; (5) Abnormal head CTP images, poor MRI quality or incomplete clinical data.

This study was approved by the Ethics Committee of Beijing Chaoyang Hospital Affiliated to Capital Medical University, and all enrolled patients gave informed consent for their clinical data to be used in the study.

### 2.2 General clinical data collection

Basic clinical data were collected, including age, sex, previous medical history (hypertension, diabetes mellitus, hyperlipidemia, stroke, and coronary heart disease), smoking, and alcohol consumption. Laboratory data included cholesterol (TC), high-density lipoprotein (HDL), low-density lipoprotein (LDL), triglycerides (TG), Lipoprotein (a), aspartate aminotransferase (AST), alanine aminotransferase (ALT), blood urea nitrogen (BUN), Creatinine (Cr), fasting blood glucose.

### 2.3 Head CTP imaging and post-processing

**Figure 1:**
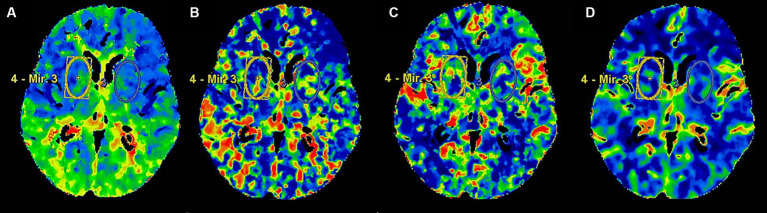
The ROI of head CTP in the basal ganglia. Panels **(A–D)** presented head CTP parameters, including TMAX, MTT, CBF and CBV, respectively.

All patients underwent a 256-slice CT scanner for head CTP imaging, CTP post-processing, and image analysis which was done on an advanced multimodal workstation using AW Server 3.2 software. After the time density curve for CTP variable calculation was generated automatically, the anterior cerebral artery and superior sagittal sinus were used as reference points, and the positioning line was turned in the axial position so that it was parallel and vertical to the brain line. Color graphs of CTP parameters including CBF, CBV, MTT and TMAX are generated using an automatic threshold-based deconvolution algorithm. Firstly, the color map was qualitatively evaluated by visual inspection for any abnormality. After the abnormal color map was excluded, a bilateral symmetrical oval region of interest (ROI) was drawn freehand in the basal ganglia region for quantitative evaluation of each perfusion parameters. According to previous studies (9, 10), the ROI of the basal ganglia was manually drawn to a standardized oval mirrored region of interest (ROI), including the caudate nucleus, putamen, external portion of the globus pallidus, internal portion of the globus pallidus, and part of subthalamic nucleus, and tried to select the same location for each measurement. The measured values of bilateral basal ganglia are obtained, and the final average value is obtained. The ROI of the basal ganglia region is about 32.0 mm on the long axis and 19.0 mm on the short axis, with an average area of 496.50 mm^2^ (Figure 1).

### 2.4 Assessment of BG-EPVS and other markers of CSVD

All patients underwent a 3.0-T head MRI examination. The diagnostic criteria for CSVD were based on the standards published by the International Society for Vascular and Endovascular Neurosurgery in 2023 (11).

BG-EPVS grading method: The level with the highest number of EPVS in the basal ganglia was selected for counting. Grade 0: 0 EPVS; Grade 1: 1 to 10 EPVS; Grade 2: 11 to 20 EPVS; Grade 3: 21 to 40 EPVS; Grade 4: >40 EPVS or unable to count (12). Cases were categorized as the BG-EPVS group (EPVS graded 3–4) and the control group (EPVS graded 0, 1and 2).

Evaluation of other CSVD: lacune (11), WMH (13), CMB (14) and cerebral atrophy (15).

### 2.5 Statistical analysis

SPSS 27.0 statistical software was used to analyze the data. The Kolmogorov–Smirnov test was used to test the normality of continuous variables. The data were expressed as the mean ± SD of normally distributed variables and the median (quartile) of non-normally distributed variables. The categorical variables were expressed as frequency and percentage. Continuous variables conforming to normal distribution were compared by and *T*-test or one-way ANOVA and non-normal distribution variables by Mann–Whitney *U* test or Kruskal–Wallis *H* test. Chi-square test was used to compare categorical variables. Point-biserial correlation analysis, binary logistic regression analysis and ordered logistic regression analysis were applied to analyze the relationship between cerebral perfusion parameters and severe BG-EPVS. Odds ratio (OR) value and 95% confidence interval (CI) were calculated. *p* < 0.05 was considered to be statistically significant.

## 3 Results

### 3.1 Comparison of general clinical data and other CSVD markers between the BG-EPVS group and the control group

The comparison results of general clinical were shown in Table 1. There were statistically significant differences in hypertension (*p* = 0.040), diabetes mellitus (*p* = 0.008) and history of stroke (*p* = 0.021) between the BG-EPVS group and the control group. There were no statistical differences in age, sex, and the proportions of the following: history of hyperlipidemia and coronary heart disease, smoking and alcoholism, and the other collected clinical characteristics between the BG-EPVS group and control group.

**Table 1:** Comparison of general clinical data and other CSVD markers between the BG-EPVS group and the control group.

Characteristics	BG-EPVS group (*n* = 77)	Control group (*n* = 89)	*P*
Age (years)	73.95 ± 6.81	72.96 ± 6.74	0.347
Male, *n* (%)	51(66.2)	62(69.7)	0.636
Hypertension, *n* (%)	62(80.5)	59(66.3)	0.040
DM, *n* (%)	25(32.5)	47(52.8)	0.008
HLP, *n* (%)	20(26.0)	16(18.0)	0.212
History of stroke, *n* (%)	29(37.7)	19(21.3)	0.021
History of CHD, *n* (%)	18(23.4)	12(13.5)	0.099
Smoking, *n* (%)	27(35.1)	30(33.7)	0.854
Alcoholism, *n* (%)	18(23.4)	27(30.3)	0.314
TC, mmol/L	4.18(3.61, 5.46)	4.43(3.835, 5.19)	0.612
HDL-C, mmol/L	1.16(0.97, 1.43)	1.12(0.93, 1.37)	0.612
LDL-C, mmol/L	2.67(1.78, 3.54)	2.69(1.87, 3.51)	0.734
TG, mmol/L	1.21(0.83, 1.82)	1.58(0.97, 2.62)	0.205
Lp (a), mmol/L	11.60(7.08, 21.83)	14.9(7.7, 30.9)	0.206
AST, U/L	18.50(15.00, 26.00)	21.00(17.5, 25.00)	0.327
ALT, U/L	15.00(11.75, 20.25)	17.00(14.00, 20.00)	0.102
BUN, mmol/L	5.73(4.41, 6.83)	5.82(4.79, 7.41)	0.426
Cr, umol/L	67.05(57.23, 78.28)	68.50(58.4, 82.20)	0.520
Ua, umol/L	326.00 ± 88.43	341.44 ± 90.08	0.782
Fasting blood glucose, mmol/L	6.79(5.60, 8.85)	7.20(6.09, 12.73)	0.142
lacune, *n*	3(2, 5)	0(0, 2)	<0.001
WMH, fazekas score	3(3, 5)	2(2, 3)	<0.001
CMB, *n*	1(0, 6.5)	0(0, 0)	<0.001
Brain atrophy, *n*	1(1, 2)	1(1, 2)	0.276

Data are presented as mean ± standard deviation, median (interquartile range) or counts (%). DM, diabetes mellitus; HLP, hyperlipidemia; CHD, coronary heart disease; TC, total cholesterol; HDL-C, high density lipoprotein; LDL-C, low density lipoprotein; TG, Triglyceride; Lp (a), lipoprotein (a); AST, aspartate aminotransferase; ALT, alanine aminotransferase; BUN, blood urea nitrogen Cr; creatinine; Ua, uric acid.

The comparison of the CSVD markers between the two groups were shown in Table 1, the BG-EPVS group had more lacunes, more serious WMH, and more CMBs (*p* < 0.001). There is no difference in brain cortical atrophy between the BG-EPVS and control group (*p* = 0.276).

### 3.2 Comparison of head CTP imaging parameters in basal ganglia between the BG-EPVS group and the control group

**Figure 2:**
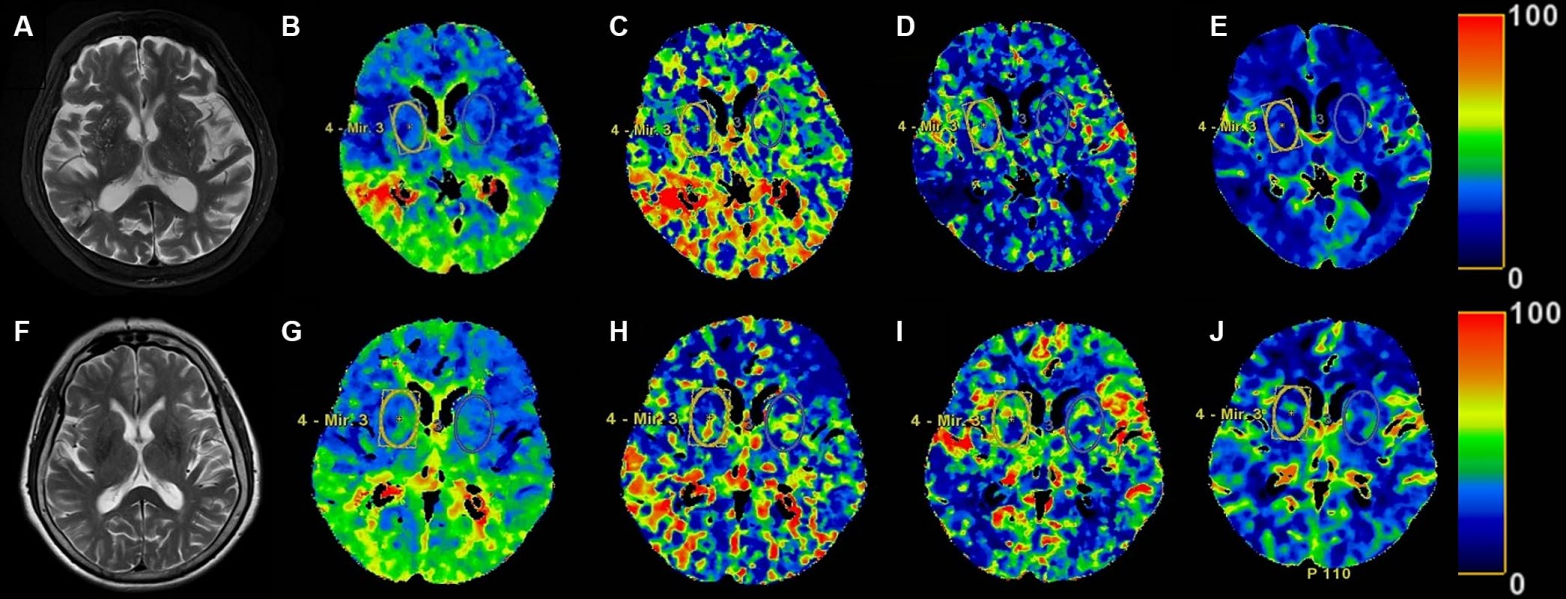
The head MRI T2 sequence images and the ROI of head CTP images in the BG-EPVS group and the control group. Panels **(A–E)**, respectively, present MRI T2 sequence, TMAX, MTT, CBF, and CBV images of the BG-EPVS group. Panels **(F–J)**, respectively, present MRI T2 sequence, TMAX, MTT, CBF, and CBV images of the control group. Panels **(E,J)** showed CBV of BG-EPVS group was lower than that of control group.

The comparison of parameters of head CTP imaging between the two groups was shown in Table 2. There were no statistical differences in TMAX, MTT and CBF between the BG-EPVS group and control group. CBV in the BG-EPVS group was significantly lower than that in the control group, with statistical significance (*p* = 0.035). Figure 2 showed the CTP images of the BG-EPVS group and the control group.

**Table 2:** Comparison of head CT perfusion imaging parameters of basal ganglia between the BG-EPVS group and the control group.

Parameters of head CT perfusion	BG-EPVS group (*n* = 77)	Control group (*n* = 89)	*p*
Tmax (s)	2.607 ± 0.893	2.606 ± 0.726	0.479
MTT (s)	5.651 ± 1.437	5.588 ± 1.240	0.846
CBF (mL/100 g/min)	29.203 ± 7.775	30.913 ± 7.611	0.155
CBV (mL/100 g)	2.204 ± 0.320	2.334 ± 0.465	0.035

### 3.3 The association between severe BG-EPVS and CBV

Point-biserial correlation analysis showed a negative correlation between BG-EPVS and CBV, indicating that as BG-EPVS grade increases, CBV tends to decrease (*r* = −0.198, *p* = 0.011). To identify the effect of CBV on severe BG-EPVS, the BG-EPVS group and control group as dependent variables, binary logistic regression was used to analyze the variables of difference between the single factor groups, and the results showed that there was no correlation between CBV and severe BG-EPVS (*p* = 0.063) after adjusting for gender and previous medical history (including hypertension, diabetes mellitus, and history of stroke) (Model 1, shown in Table 3). After adjusting for all confounders (Model 2), including sex, previous medical history (including hypertension, diabetes mellitus, and history of stroke), and other types of CSVD (lacune, WMH fazekas score, CMB, and brain atrophy), there was no association between CBV and BG-EPVS severity (*p* = 0.448).

**Table 3:** Binary logistics regression of independent risk factors for BG-EPVS.

	Model 1				Model 2			
	*B*	OR	95%CI	*P*	*B*	OR	95%CI	*P*
CBV (mL/100 g)	−0.779	0.459	0.202–1.042	0.063	−0.804	0.426	0.175–1.146	0.448

Model 1: binary logistics regression analysis and adjustment for hypertension, diabetes and stroke history; Model 2: adjusted for all variables, including hypertension, diabetes mellitus, history of stroke, the number of lacune and CMB, and the WMH Fazekas score.

In addition, all enrolled patients were divided into four groups according to the interquartile interval of CBV. The first group was CBV distributed in the range of 75–100%, with 46 people; the second group was CBV distributed in the range of 50–74%, with 37 people; the third group had CBV in the range of 25–49%, with 42 people; the fourth group had a CBV in the range of 0–24%, with 41 people. The results of comparison of general clinical data are shown in Table 4. There were statistically significant differences in gender (*p* = 0.047), history of hyperlipidemia (*p* = 0.040), smoking (*p* = 0.036), and alcoholism (*p* < 0.001) in CBV group 1, CBV group 2, CBV group 3, and CBV group 4. There were no significant differences in age, hypertension, diabetes, history of stroke and coronary heart disease, other clinical features collected, and other cerebral small vessel disease markers among the groups. There were statistically significant differences in the severity of BG-EPVS among CBV group 1, CBV group 2, CBV group 3, and CBV group 4 (*p* = 0.047).

**Table 4:** Comparison of general clinical data and other CSVD markers of all enrolled patients based on basal ganglia cerebral blood volume (CBV) interquartile spacing.

Characteristics	CBV Group 1 (*n* = 46)	CBV Group 2 (*n* = 37)	CBV Group 3 (*n* = 42)	CBV Group 4 (*n* = 41)	*P*
Age (years)	72.0(67.75, 78.0)	72.0(64.0, 79.5)	73.5(70.25, 78.0)	73.0(70.5, 76.5)	0.798
Male, *n* (%)	27(58.70)	24(64.86)	27(64.29)	35(85.37)	0.047
Hypertension, *n* (%)	34(73.91)	26(70.27)	32(76.19)	29(70.73)	0.923
DM, *n* (%)	25(54.35)	13(35.14)	15(35.71)	19(46.34)	0.219
HLP, *n* (%)	9(19.57)	14(37.84)	5(11.90)	8(19.51)	0.040
History of stroke, *n* (%)	12(26.09)	13(35.14)	8(19.05)	15(36.59)	0.257
History of CHD, *n* (%)	10(21.74)	4(10.81)	7(16.67)	9(21.95)	0.530
Smoking, *n* (%)	12(26.09)	8(21.62)	17(40.48)	20(48.78)	0.036
Alcoholism, *n* (%)	8(17.39)	5(13.51)	11(26.19)	21(51.22)	<0.001
TC, mmol/L	4.67 ± 1.41	4.31 ± 1.04	5.02 ± 1.31	4.32 ± 0.97	0.159
HDL-C, mmol/L	1.15 ± 0.38	1.25 ± 0.36	1.22 ± 0.41	1.21 ± 0.34	0.678
LDL-C, mmol/L	2.83 ± 1.22	2.61 ± 0.90	3.00 ± 1.18	2.65 ± 0.98	0.584
TG, mmol/L	2.20 ± 1.58	1.45 ± 0.80	2.72 ± 3.17	1.81 ± 1.79	0.348
Lp (a), mmol/L	28.52 ± 36.50	30.07 ± 32.76	16.27 ± 17.70	16.99 ± 14.81	0.109
AST, U/L	25.20 ± 14.04	21.07 ± 6.30	19.28 ± 5.50	22.68 ± 7.20	0.389
ALT, U/L	21.66 ± 15.98	17.43 ± 7.55	19.68 ± 18.90	17.52 ± 5.75	0.766
BUN, mmol/L	6.44 ± 1.81	6.09 ± 3.21	6.05 ± 2.23	5.95 ± 1.79	0.853
Cr, umol/L	70.75 ± 20.95	78.90 ± 38.53	63.70 ± 14.53	71.14 ± 12.42	0.500
Ua, umol/L	356.12 ± 94.37	316.10 ± 75.65	306.16 ± 98.66	356.06 ± 79.77	0.157
Fasting blood glucose, mmol/L	9.03 ± 4.71	7.66 ± 3.78	11.54 ± 5.20	7.97 ± 2.94	0.139
lacune, *n*	1(0, 3)	1(1, 2)	2(1, 4.25)	1(0, 3)	0.122
WMH, fazekas score	3(2, 4)	3(2, 3)	3(2, 4)	3(2, 4.5)	0.478
CMB, *n*	0(0, 1)	0(0, 1)	0(0, 1)	0(0, 1)	0.474
Brain atrophy, *n*	1(1, 2)	1(1, 1)	1(1, 2)	1(1, 2)	0.054

Data are presented as mean ± standard deviation, median (interquartile range) or counts (%). DM, diabetes mellitus; CHD, coronary heart disease; HbA1c, glycosylated hemoglobin; HLP, hyperlipidemia; TC, total cholesterol; HDL-C, high density lipoprotein; LDL-C, low density lipoprotein; TG, Triglyceride; Lp (a), lipoprotein (a); AST, aspartate aminotransferase; ALT, alanine aminotransferase; BUN, blood urea nitrogen Cr; creatinine; Ua, uric acid.

To identify the effect of BG-EPVS on CBV, we took the four groups divided by interquartile interval of CBV as dependent variables and performed ordered logistics regression analysis. The results were shown in Table 5. After adjusting for gender, history of hyperlipidemia, smoking and drinking, ordered logistic regression analysis showed that severe BG-EPVS was independent risk factor decreased CBV (*p* = 0.009), and we also found that there was a significant correlation between alcohol consumption and decreased CBV (*p* = 0.004). In addition, we evaluated whether the severe BG-EPVS effect was different between men and women. The result showed that there was no difference in BG-EPVS effect between men and women.

**Table 5:** Ordered logistics regression of the correlation between CBV and BG-EPVS.

	*B*	OR	95%CI	*P*
Male	0.495	1.583	0.819–3.060	0.172
HLP	0.011	1.011	0.501–2.039	0.976
Smoking	0.072	0.930	0.426–2.030	0.856
Alcoholism	1.250	3.490	1.499–8.126	0.004
BG-EPVS	0.762	2.142	1.211–3.788	0.009

All enrolled patients were divided into four groups according to the interquartile interval of CBV. HLP, hyperlipidemia.

## 4 Discussion

In this study, we explored the association between severe BG-EPVS and cerebral perfusion in elderly individuals by head CTP. The cerebral perfusion parameters included TMAX, MTT, CBF and CBV. We found that severe BG-EPVS was an independent risk factor for decreased CBV in basal ganglia after adjusting for other CSVD markers.

The results of current research on the relationship between CSVD and cerebral perfusion are inconsistent. Although some studies have shown that CBF is related to the increase of CSVD load, other studies have shown that CBF is unrelated to the total CSVD load or various CSVD types (16). The latter view is consistent with our findings that CBF and CBV are not related to the severity of BG-EPVS. Bastos-Leite et al. (17) utilized Pulsed Arterial Spin-Labeling (PASL) to investigate the correlation between CBF and WMH, revealing a notable decrease in CBF among elderly individuals with diffuse confluent WMH. Hashimoto et al. (6) showed a link between increased numbers of CMB and cerebral ischemia in deep white matter patients. Mochizuki et al. (7) observed reduced blood flow in lacune infarctions. To the best of our knowledge, this is the first study to examine the association between BG-EPVS and cerebral perfusion by head CTP. Our findings indicate that CBV did not emerge as a risk factor for severe BG-EPVS, however, severe BG-EPVS is an independent risk factor for decreased CBV after controlling for confounding factors.

The potential pathophysiological mechanisms underlying the association between large numbers of BG-EPVS and CBV are not completely understood. EPVS is surrounded by small arteries and veins. An increased permeability of the small vessel walls and blood–brain barrier is considered to contribute to the development of EPVS, which has been reported to be associated with damage of microvascular endothelial cells and their tight junctions. In addition, leakage of interstitial fluid may cause obstruction of drainage space and lead to occurrence of EPVS (18). When perivascular space expands, there is a close relationship with cerebral blood flow, which involves the interaction of multiple physiological and pathological factors. Based on the results of this study, we speculate that the potential pathophysiological mechanisms may include the following aspects. Firstly, the BG-EPVS may be associated with local inflammation and injury (19–21), resulting in reduced cerebral blood flow. Inflammation and injury may cause changes in the perivascular tissue, including widening of the space and increased local vascular permeability. In this case, immune cells and inflammatory mediators may affect hemodynamics, resulting in decreased cerebral blood volume. Secondly, the BG-EPVS may be related to changes in the blood–brain barrier, which may lead to reduced cerebral blood flow. The blood–brain barrier is an important physiological barrier that maintains the stability of the internal and external environment of brain tissue. When the perivascular space expands, the integrity of the blood–brain barrier may be damaged (22), resulting in some harmful substances entering the basal ganglia more easily, negatively affecting its function. This may include thrombosis, infiltration of neurotoxic substances (23), which may lead to reduced cerebral blood flow and thus affect cerebral blood volume. In addition, the BG-EPVS may be directly related to reduced cerebral blood flow. The expansion of PVS may lead to the increase of vascular permeability (19), which will affect the blood perfusion in this area and thus affect the cerebral blood volume.

In this study, we found that hypertension, diabetes, and stroke history were associated with an increased burden of EPVS. Some studies have shown that hypertension is closely related to BG-EPVS. The mechanism may be that long-term hypertension leads to injury of cerebral small blood vessel endothelial cells, increased permeability of blood vessel wall, excessive leakage of intravascular substances and accumulation in PVS, causing expansion of PVS and eventually forming EPVS (24–26). Recent studies have also found diabetes is associated with EPVS. Attenuation and/or loss of the brain endothelial cell glycocalyx in diabetic patients lead to leads to breakdown of the brain barrier, increased permeability of pro-inflammatory white leukocytes, fluids, and solutes, and accumulation in the space around the postcapillary venule perivascular spaces. This accumulation results in obstruction and results in EPVS (27, 28). Some studies have found that stroke can lead to the increase of EPVS, which is related to the hypoxia and injury of local brain tissue and the increase of the permeability of the blood–brain barrier, so that the components of blood enter the brain tissue, causing inflammation and edema which further contributes to the development of EPVS (29–31).

Our results provide some new insights. Firstly, there is a significant negative correlation between the severity of BG-EPVS and CBV. This may be related to the abnormal microcirculation in the basal ganglia caused by factors such as inflammation, destruction of the blood–brain barrier, and increased permeability of the vascular wall, which in turn affect the cerebral blood volume of the surrounding brain tissue. Secondly, interestingly, we did not observe a correlation between the severity of CBV and the severity of BG-EPVS. This means that hemodynamic changes are not the only factor contributing to the formation of BG-EPVS, and other factors such as neuromodulation in the basal ganglia may also be involved. Our study provides important clues to understanding the relationship between BG-EPVS and CBV, but more in-depth research is still needed to elucidate the specific mechanisms of this relationship. Future studies can further clarify the formation mechanism of BG-EPVS, thereby enhancing our understanding of its development and impact in the brain.

There are some limitations to our study. Firstly, our study population was based on hospital patients in a single center. Secondly, it is too difficult to place the ROI completely on the basal ganglia area, because the size of the basal ganglia area varies from person to person, and there is no guarantee of full inclusion. We have done our best to put ROI on the basal ganglia, but there may still be some heterogeneity. Finally, this was a case–control study, and future longitudinal studies are needed to verify the causal relationship between CBV and EPVS severity. In a word, there is a complex and close relationship between BG-EPVS and cerebral perfusion. This relationship involves the interaction of multiple pathophysiological factors, which may include vascular permeability, inflammation, and the blood–brain barrier. There is a significant negative correlation between the severity of BG-EPVS and CBV, and the formation of BG-EPVS is not solely dependent on changes in cerebral blood volume in this region.

In conclusion, in the present study, we found severe BG-EPVS was an independent risk factor for decreased CBV by CTP in older adults and the formation of BG-EPVS was not solely dependent on changes in CBV in this region. This finding provides information about the pathophysiological consequence caused by severe BG-EPVS.

## Data availability statement

The raw data supporting the conclusions of this article will be made available by the authors, without undue reservation.

## Ethics statement

The studies involving humans were approved by Beijing Chao-Yang Hospital, Capital Medical University, Beijing, China. The studies were conducted in accordance with the local legislation and institutional requirements. The participants provided their written informed consent to participate in this study. Written informed consent was obtained from the individual(s) for the publication of any potentially identifiable images or data included in this article.

## Author contributions

SW: Data curation, Writing – original draft, Investigation. SY: Conceptualization, Data curation, Funding acquisition, Writing – review & editing. DL: Formal analysis, Writing – review & editing. WQ: Project administration, Writing – review & editing. LY: Project administration, Writing – review & editing. XL: Project administration, Writing – review & editing. WH: Conceptualization, Methodology, Supervision, Writing – review & editing.
